# Realistic Three Dimensional Fitness Landscapes Generated by Self Organizing Maps for the Analysis of Experimental HIV-1 Evolution

**DOI:** 10.1371/journal.pone.0088579

**Published:** 2014-02-28

**Authors:** Ramón Lorenzo-Redondo, Soledad Delgado, Federico Morán, Cecilio Lopez-Galindez

**Affiliations:** 1 Centro Nacional de Microbiología, Instituto de Salud Carlos III, Majadahonda, Madrid, Spain; 2 Departamento de Organización y Estructura de la Información, Escuela Universitaria de Informática, Universidad Politécnica de Madrid, Madrid, Spain; 3 Departamento de Bioquímica y Biología Molecular I, Universidad Complutense de Madrid, Madrid, Spain; Institut Pasteur, France

## Abstract

Human Immunodeficiency Virus type 1 (HIV-1) because of high mutation rates, large population sizes, and rapid replication, exhibits complex evolutionary strategies. For the analysis of evolutionary processes, the graphical representation of fitness landscapes provides a significant advantage. The experimental determination of viral fitness remains, in general, difficult and consequently most published fitness landscapes have been artificial, theoretical or estimated. Self-Organizing Maps (SOM) are a class of Artificial Neural Network (ANN) for the generation of topological ordered maps. Here, three-dimensional (3D) data driven fitness landscapes, derived from a collection of sequences from HIV-1 viruses after “in vitro” passages and labelled with the corresponding experimental fitness values, were created by SOM. These maps were used for the visualization and study of the evolutionary process of HIV-1 “in vitro” fitness recovery, by directly relating fitness values with viral sequences. In addition to the representation of the sequence space search carried out by the viruses, these landscapes could also be applied for the analysis of related variants like members of viral quasiespecies. SOM maps permit the visualization of the complex evolutionary pathways in HIV-1 fitness recovery. SOM fitness landscapes have an enormous potential for the study of evolution in related viruses of “in vitro” works or from “in vivo” clinical studies with human, animal or plant viral infections.

## Introduction

**Figure 1:**
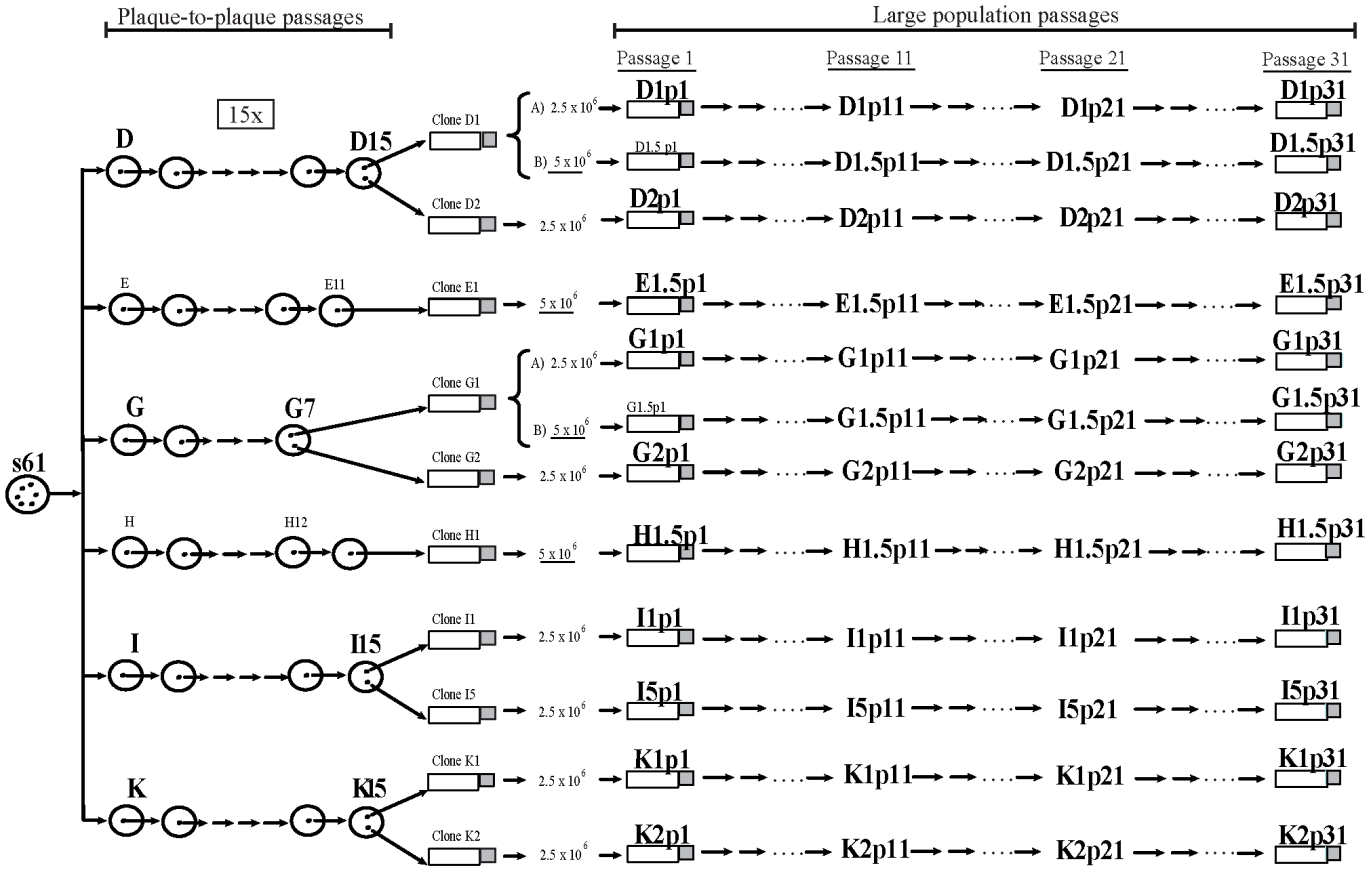
Genealogy of the HIV-1 viral clones studied. Schematic representation of the serial passages performed with the viruses. Six biological clones, derived from a natural isolate [3], represented by circles in the left part of the Figure, were plaque to plaque passaged for 15 rounds resulting in drastic fitness losses [4]. Some of the clones (*G*, *E* and *H*) did not overcame the 15 passages [4]. In general, two clones from the final debilitated biological clones designated *D1*, *D2*, *E1*, *G1*, *G2*, *H1*, *I1*, *I5*, *K1* and *K2* were later subjected to large population recovery passages [6]. Large population passages (10, 20 and 30) with these clones, represented by bottles, arrows and dots in the right part of the figure, were performed in 2.5×10^6^ and 5×10^6^ MT-4 cells [6]. Viral populations are indicated by letters identifying the clone, followed by p1 for the initial population, p11 for passage 11, p21 for passage 21 and p31 for passage 31 [6], [7]. Clones *D1* and *G1* that are represented after keys were passaged in parallel in 2.5×10^6^ (designated A) and in 5×10^6^ MT-4 cells (designated B) [7]. Clones *E1*, and *H1* were passaged only in 5×10^6^ MT-4 cells. The set of 55 viruses used in the present work are marked in bigger and bold font.

**Figure 2:**
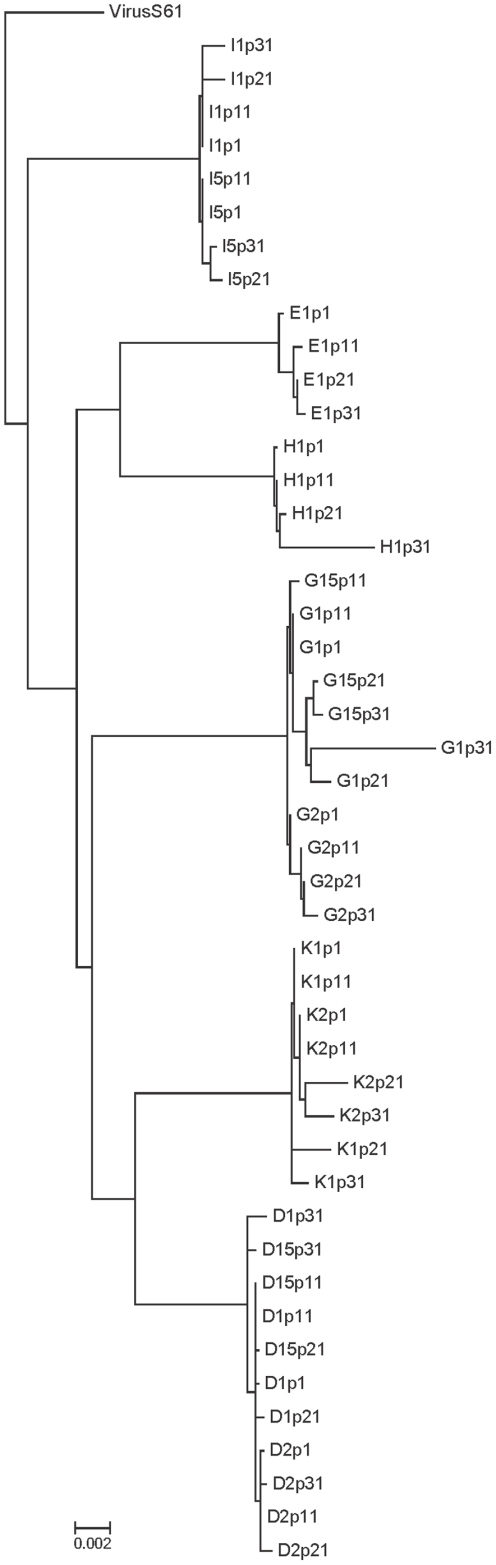
Maximum Likelihood phylogenetic tree of the studied viruses. Maximum Likelihood tree constructed with the complete genomic sequences of the studied viruses and the parental *S61* virus. The tree parameters of the weighted evolutionary model were obtained previously by JModelTest and the tree was obtained by the PHYML program. Viruses grouped by lineages with some long branches. Bar represents the genetic distance.

Human Immunodeficiency Virus type 1 (HIV-1) is characterized by high mutation rates, large population sizes, and rapid replication rates. As a result, HIV-1 exhibits complex evolutionary strategies [1], [2]. HIV-1 “*in vitro*” studies after serial culture passages with alterations in the population size have been used for the simulation and study of viral evolution (Figure 1 and 2) [3]–[7]. These studies permitted the investigation of the fitness recovery, the dynamics of viral quasispecies and the overall viral evolution [3]–[8].

Fitness landscapes, a graphic representation of evolutionary processes, togheter with mutations rates and population sizes gives an approach for the study of evolution [9]. For the investigation on the evolution of organisms, Sewall Wright [9] depicted the change in allele frequency for the production of adaptative landscapes. These landscapes were primarily used for the illustration of evolutionary pathways. Afterwards, they were extended with the concept of sequence space [10] and for the generation of fitness landscapes [11], [12]. These maps are widely used for the analysis of evolution in different organism from vertebrates [13] to bacteriophages [14], [15], RNA viruses [16]–[18], including HIV-1 [19]–[21], to small RNAs [22]–[25] or proteins [26]. In addition, fitness landscapes make possible the investigation of evolutionary pathways. In general, the direct experimental determination of real fitness values in organisms is difficult [27], and its detection is restricted to limited alleles [28]. In consequence, most fitness landscapes have been artificial, theoretical or estimated [13] and realistic fitness landscapes are lacking [28].

The study of evolution in small RNA artifitial populations was performed, several years ago, by the projection of sequence data from high-dimensional sequence space into two dimensions, using the two largest eigenvectors to obtain the bi-dimensional sequences coordinates [29], [30]. The connection of sequences was performed by the computation of the minimum spanning tree by means of a tree with the minimum total cost [29], [30]. Self-Organizing Map (SOM) is a class of Artificial Neural Network (ANN) that provides the projection of a high-dimensional input space on a two-dimensional topologically ordered map. The map produced by the SOM training algorithm [31] is formed by a set of reference vectors also known as neurons, organized in a bi-dimensional grid. The training algorithm adjusts the values of the reference vectors (nucleotide sequences in this work) through an iterative process that uses a set of sample vectors of the input space. As the relatedness of viruses in the network is a consequence of the sequence composition, the experimental fitness determination of some viruses could allow, in a non-probabilistic manner, the association in the SOM map of a fitness value with a viral sequence.

The objective of this work is the study of the biological fitness recovery of HIV-1 viral populations after “*in vitro*” culture. To this end, data driven three-dimensional (3D) fitness landscapes have been created with HIV-1 experimental data, using Self Organizing Maps (SOM) [31]. These fitness landscapes were also employed for the visualization and study of fitness in related members of viral quasispecies from the recovered viral populations and the depiction of recovery pathways.

## Materials and Methods

### Origin of the HIV-1 biological clones studied

The biological clones of the study (D, E, G, H, I, and K), derived from a natural isolate [3], were subjected to different serial passages (Figure 1). They were first plaque to plaque passaged for 15 rounds in MT-4 cells with important fitness losses [4]. From these final populations, in general, two biological clones were obtained. These clones were subjected to 30 serial large population recovery passages in MT-4 cells resulting in a progressive increase in viral fitness [5]–[7]. These serial passages were performed by infecting 2.5 or 5×10^6^ MT-4 cells (Figure 1). Clones D1 and G1 were passaged in parallel in 2.5 and 5×10^6^ MT-4 cells and designated A and B in Figure 1. Viruses were recovered from the culture supernatant at 5 to 7 days post-infection, and used for the inoculation of the following passage [3]. All these experiments produced a set of 55 viruses (Figure 1). In every virus from this set, a virological characterization was performed, in previous studies of the laboratory, including the p24 production and viral titer (Table S1 in File S1) [4], [6], [7] and Lorenzo-Redondo et al (manuscript in preparation). p24 viral production was measured with the Elecsys (HIV Ag, Roche) and viral titer was performed in MT-2 cells and expressed as tissue culture infecting dose 50 (TCID50/ml) (Table S1 in File S1). Viral fitness was calculated in competition cultures against a common virus [4], [6], [7] and Lorenzo-Redondo et al. (manuscript in preparation). DNA extraction, complete genome sequencing, fitness assay, and GeneScan quantification have been previously described [6], [7]. Viral divergence was calculated as the mean evolutionary divergence expressed as the number of bases substitutions per site (in percentage) ± standard error, using the Maximum Composite Likelihood model as in [32]. For simplicity, in Table 1 of the manuscript are shown the mean divergence between lineages, while in Table S2 in File S1 is presented the complete set of genetic distances. A phylogenetic analysis by the Maximum-Likelihood method was carried out to study the genetic relationship and variability of the viruses (Figure 2).

**Table 1:** Mean genetic divergence between lineages.

Lineages	D		E		G		H		I	
	distance^a^	error^b^	distance^a^	error^b^	distance^a^	error^b^	distance^a^	error^b^	distance^a^	error^b^
D										
E	1.0	0.1								
G	1.4	0.1	1.4	0.1						
H	1.3	0.1	1.2	0.1	1.3	0.1				
I	1.0	0.1	1.2	0.1	1.6	0.1	1.5	0.1		
K	1.0	0.1	1.4	0.1	1.4	0.1	1.2	0.1	1.4	0.1

a Estimates of evolutionary divergence between lineages expressed as mean number of base substitutions per site in sequence pairs.

b Standard error estimate(s). Analyses were conducted using the Maximum Composite Likelihood model [32]. The analysis involved 46 nucleotide sequences and a total of 8663 positions in the final dataset. Codon positions included were 1st+2nd+3rd+Noncoding. All positions containing gaps and missing data were eliminated. Evolutionary analyses were conducted in MEGA5program [32].

### Quasispecies analysis

For the analysis of the mutant spectra of the recovered viruses , we used four different genomic regions [6], [7]. In the present work, we selected only one of the regions, the V1–V2 region, due to its importance in viral infectivity and tropism. For this analysis, we amplified a fragment of 690 nucleotides, from positions 6045 to 6735, emcompassing from *vpu* to the V1–V2 region in *env*. This fragment was divided in two regions one corresponding to the *vpu* gene and the other to the V1–V2 *env* gene [7]. The V1–V2 quasispecies sequences were processed by SOM to check the usefulness of the method to approach fitness values, based on sequence similarity, and for the prediction of the complex evolutionary pathways followed by the viruses in the fitness recovery.

### SOM

SOM algorithm topologically orders data of high dimension, by an unsupervised process, for the creation of a two-dimensional grid of reference vectors. For the specific case of the viral sequences of this study, the SOM algorithm generated an ordered grid in which each node (neuron) was associated with a reference DNA sequence (see SOM parameters used in Table S3 in File S1). Each neuron of the network maps all input sequences with a distance to its reference vector smaller than the rest of reference vectors. This distance is calculated by an innovative nucleotide codification method (see nucleotide codification method in File S2). The entire set of training sequences can be projected by the trained SOM, producing a two-dimensional map ordered by similarity between the training set of DNA sequences (see Figure S1 in File S2). The trained SOM can also be used for the projection of DNA sequences not employed during the training, and the sequences will map to the neuron with the closest reference vector. In this way, a two-dimensional graph was produced, showing similarity relationships based, exclusively, on the sequence information of the DNA chains. It is important to highlight that the training and new sequences must have the same length. Furthermore, new sequences projected on the SOM should have similarity with those used for training. This is because the knowledge acquired by the SOM is based on the information in the training sequences, and thus, SOM representation is bounded to the domain defined by the training sequences.

In the SOM trained with DNA sequences, a three-dimensional map can be constructed labelling each neuron with the value of a property not used in the training. In this study, the labelling of the SOM map was carried out with the experimental fitness values [4], [6], [7] and Lorenzo-Redondo et al. (manuscript in preparation). This tagging required a set of DNA sequences from viruses with its fitness calculated from competition experiments in the laboratory [6], [7]. Each neuron of the SOM was labelled with the weighted average value of the fitness corresponding to the *L* DNA sequences of this set, closest to the reference vector of the neuron [33]. For the calculation of the fitness value of a neuron, the fitness associated to the *L* DNA sequences is weighted by the distance with the sequence of the reference vector of the neuron. Thus, the *L* parameter determines the number of different DNA sequences used for the labelling. The higher the *L* value, the higher the number of sequences is used in fitness information, and the landscape is smoother. Using *L* = 1, the fitness label of a neuron corresponds to the fitness value of the closest DNA sequence.

The three dimensional (3D) map created by the SOM is a graphical visualization of the fitness landscape associated with the sequences employed in SOM training and labelling. When DNA sequences present sequential mutations within viral populations, potential evolutionary pathways can be displayed on the 3D map, as well as topographical characteristics of the region can be defined, like abrupt areas, deep valleys or flat regions. The software used in this work can be requested in sole@eui.upm.es, and was developed for the Ph.D http://oa.upm.es/1930/.

## Results

### Virological characterization of the related HIV-1 clones

Six biological clones (*D*, *E*, *G*, *H*, *I*, and *K*) derived from isolate *s61* were subjected to “*in vitro*” serial passages (see Materials and Methods and Figure 1) generating a collection of 55 viruses (Figure 1). In every virus from this set viral production, measured by 24 levels and viral titers were determined (Table S1). Using competition cultures against the same reference virus, we calculated the fitness of every virus (Table 2) [4]–[7]. In the initial viruses, fitness values were between 0.2 in viruses from lineage *D* and 0.7 in virus *I1*. After the passages, the maximum fitness values raised to 3.4 in *G1p31* or to 2.8 in *H1.p31* (Table 2). Others viruses had more moderate but constant increases like *E1.5* virus, and two viruses (*D1.5* and *G1.5*) had fitness decreases at passage 31 (Table 2); finally, one of the virus (*K2*) was not able, after the passages, to increase fitness. In summary, during the recovery passages, although uneven in magnitude and among lineages (Table 2), there was a global increase in viral fitness.

**Table 2:** Fitness values of the viruses and their increases during the recovery passages.

	Passage							
	1	11		21		31		
Virus	Mean^a^	Mean	Increase(X)	Mean	Increase(X)	Mean	Increase(X)	Total Increase^b^
**D1**	0.2±0.13	0.9±0.06	4.8×	1.05±0.05	1.15×	1.5±0.3	1.42×	4.8×
**D1.5**	0.2±0.13	0.9±0.07	4.8×	1.4±0.24	1.51×	0.8±0.27	0.6×	0.7×
**D2**	0.3±0.03	1.00±0.02	3.5×	1.00±0.01	1×	1.5±0.24	1.6×	5.4×
**E1.5**	0.65±0.04	0.85±0.03	1.3×	0.9±0.05	1.03×	0.9±0.08	1.04×	1.6×
**G1**	0.6±0.05	0.8±0.03	1.2×	1.3±0.18	1.7×	3.4±1.77	2.6×	5.5×
**G1.5**	0.6±0.05	0.7±0.02	1.1×	1.2±0.2	1.7×	0.5±0.04	0.38×	0.75×
**G2**	0.7±0.03	0.7±0.01	1.02×	0.8±0.08	1.07×	1.7±0.38	2.2×	2.5×
**H1.5**	0.5±0.04	0.95±0.01	1.9×	1.6±0.15	1.6×	2.8±1.58	1.8×	5.5×
**I1**	0.5±0.09	0.7±0.02	1.3×	0.8±0.01	1.2×	1.9±0.25	2.3×	3.5×
**I5**	0.6±0.03	0.6±0.03	0.9×	0.9±0.15	1.6×	1.2±0.47	1.3×	2×
**K1**	0.7±0	0.8±0	1.2×	0.8±0.02	0.94×	2.00±1.07	2.6×	2.9×
**K2**	0.7±0.04	0.7±0.04	0.98×	0.7±0.05	1	0.6±0.27	0.94	0.92

a Mean fitness values ± standard error. Fitness values were calculated in competition cultures against a common reference virus as described in [4], [6], [7].

b Total increase refers to the fold increase between the initial and the final passage 31 populations.

The complete global nucleotide sequence from all recovered viruses [6], [7] was obtained for the study of the accumulation of mutations in the fitness recovery as well as for the evolution of the different lineages. Viral divergence between viruses, calculated as described in Materials and Methods, was up to a maximum mean distance between lineages of 1.6% (Table 1) and a maximum of 2.1% between individual viruses *G1p31* and *I1p21* (Table S2) [32]. Estimation of the phylogenetic relationships between the viruses was carried out by Maximum Likelihood (ML) method and the corresponding tree is shown in Figure 2. In general, there were no branches in the tree showing high evolutionary distances, except in *K* and *D* lineages in passage 21 and 31, and the two branches with the highest estimated distances that corresponded with viruses with the largest fitness increases (*G1p31* and *H1p31*, Figure 2 and Table 2). The phylogenetic tree permitted the analysis of viral evolution and the display of the clustering of lineages.

Quasispecies analysis in the viral populations was carried out to study the viral diversification along passages. This quasispecies analysis was performed by examination of 20 clones per sample during the recovery passage (Table 3) in the V1–V2 region in *env* gene which is an important region for viral fitness (Materials and Methods). Viruses with important fitness increases (see for example viruses *G1p31* and *H1p31*) showed the highest gain in quasispecies heterogeneity in passage 21 and 31, whereas viruses with limited increase in heterogeneity showed limited increase in fitness (viruses *E1.5*). Thus, increases in viral heterogeneity correlated significantly with the observed increases in viral fitness (Table 2) [7].

**Table 3:** Quasispecies diversity in the V1–V2 region in *env* gene of the recovered viruses.

	Passages			
	1	11	21	31
Virus	Mean^a^	Mean^a^	Mean^a^	Mean^a^
D1	0,36±0.23^a^	0,30±0.17	0,22±0.20	0,34±0.34
D1.5	0,36±0.23	0,38±0.13	0,15±0.11	0,20±0.16
D2	0,17±0.16	0,31±0.25	0,44±023	0,34±0.11
E1.5	0,38±0.24	0,44±0.22	0,32±0.13	0,38±0.16
G1	0,25±0.17	0,36±0.18	0,84±0.40	0,63±0.23
G1.5	0,25±0.17	0,50±0.02	0,26±0.18	0,28±0.16
G2	0,28±0.20	0,47±0.27	0,69±0.50	0,11±0.10
H1.5	0,30±0.21	0,46±0.20	0,77±0.27	0,58±0.19
I1	0,22±0.02	0,31±0.16	0,55±0.21	0,67±0.47
I5	0,16±0.16	0,24±0.15	0,36±0.17	0,49±0.32
K1	0,26±0.22	0,40±0.15	0,39±0.14	0,49±0.24
K2	0,33±0.03	0,34±0.18	0,48±0.30	0,50±0.21

a Mean genetic distance measured as substitutions per site in all pairwise comparison. As heterogeneity differences between the four regions studied were minor, we used the mean genetic distance of all viruses. The quasispecies heterogeneity was estimated using the mean genetic distance of the nucleotide sequences by Maximum Likelihood after the use of the jModeltest to establish the parameters which selected the GTR+G model. The estimation was carried with the PAUP program [40].

### SOM landscapes

**Figure 3:**
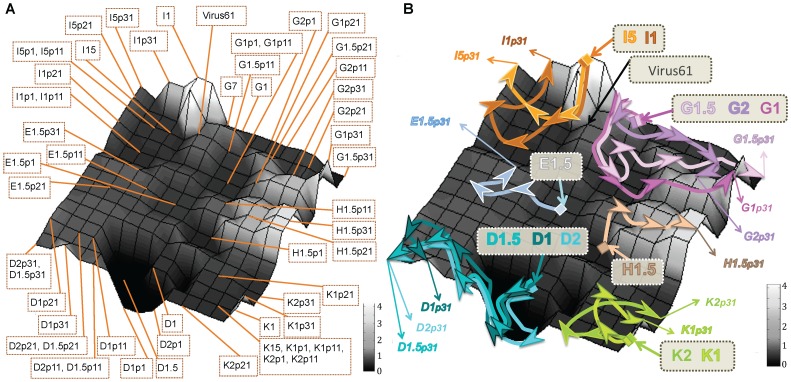
Representation of the fitness landscape of the HIV-1 studied viruses from the complete genome sequences and depiction of the viral recovery pathways. The landscape was constructed by SOM with the complete genomic sequences from the set of 55 viruses (see Figure 1) and labelled with the fitness value of the closest sequence (factor *L* = 1 was used to label the network). The SOM was formed by a grid of 15×15 neurons (Fig. S1). Each vertex of the bi-dimensional mesh symbolized a neuron of the SOM network. Grey scale of the landscape represents the fitness values, from the lowest values in black to the highest in white. A) Fitness landscape showing the neuron that maps each viral sequence. B) Fitness landscape map displaying the pathways followed by the different viruses during the recovery passages, where the viruses from the same lineage are linked with the same colour arrow.

To further investigate the evolution of these “*in vitro*” viruses and to study the relationship between nucleotide sequence and fitness, we exploited the potential of the SOM algorithm, for the fitness landscape representation during fitness recovery. The first analysis was performed training a SOM network with the complete viral nucleotide sequences of the 55 viruses highlighted in Figure 1 and as detailed in Materials and Methods. As a result of the SOM algorithm, each viral sequence mapped in a two-dimensional lattice by means of sequence similarity (Figure S1 in File S2). Later each neuron in the SOM grid was labelled as a third dimension with the average value of the experimental fitness corresponding to the closest *L* sequences , in Euclidean distance, to its reference vector (Materials and Methods) [4]–[7], [33]. The resulting fitness landscape classified the studied viruses, and presented a rugged topology with diverse peaks and valleys (Figure 3A). As in the phylogenetic tree, viruses of the same lineage clustered together in the topological ordering performed by the SOM (Figure 2). Comparing the experimental fitness value of each viral sequence with the fitness value associated with the neuron that maps the sequence on the SOM, a 0.96 Pearson correlation coeficient was obtained (Figure S2 in File S2). The topological assembling of the sequences, along with the high correlation value confirms that the 3D fitness landscape obtained by the SOM is a realistic graphical representation of the sequences and the experimental fitness associated with the viruses.

This fitness landscape identified abrupt zones, like peaks (*I* lineage) or deep fitness valleys where, for example, lineage *D* viruses, the viruses with the lowest initial fitness (Table 2), were located. Using the SOM fitness landscape, the pathways followed by viruses during the passages of fitness recovery could be tracked and represented as shown by the colour arrows in Figure 3B. Viruses from lineages *G* and *K* mapped in peaks, where the accumulation of mutations during the recovery passages led to long and irregular trajectories of fitness increase. On the contrary, constant regions of the landscape like those where *H* and *E* viruses mapped were also identified, and these viruses recovered fitness with a more regular pattern and lower risk of deleterious mutations. The virus with the highest initial fitness value, clone *I* mapped in the highest fitness peak of the landscape.

Analysis of the recovery pathways in the fitness landscape showed that viruses with a more limited space search had, in general, more limited fitness gains (see viruses *E1* and *I5* in Tables 1 and 2 and Figure 3B), whereas viruses that underwent a wider space search are those with larger fitness recovery (viruses *G1* and *H1* in Figure 3B). The construction by SOM of a fitness landscape with real sequence data permitted the analysis of the process of HIV-1 evolution during recovery passages and the 3D graphical representation of evolutionary pathways.

### Three dimensional SOM landscapes for analysis of related variants

**Figure 4:**
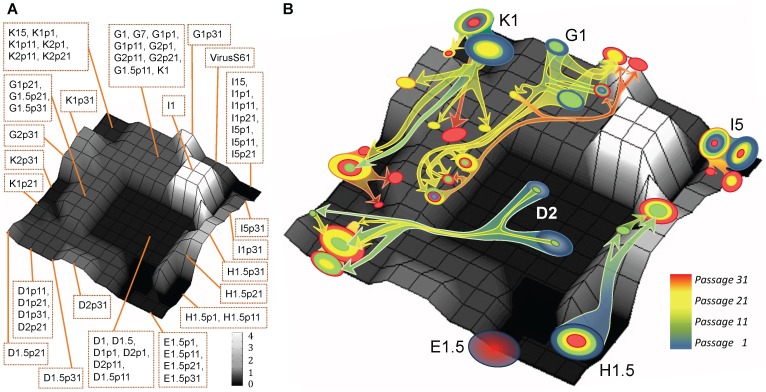
Representation of the fitness landscape from viral consensus sequences in the V1–V2 region in *env* gene and of the evolutionary trajectories of quasispecies variants. The landscape was created by SOM (15×15 neurons) with the 55 consensus sequences in the V1–V2 region in *env* gene from the global sequences, labelled as in Figure 3 (with an *L* = 1 factor) and drawn using the same grey scale as in Figure 3. A) Fitness landscape map showing the neuron that maps each viral consensus sequence. B) Representation of some of the 911 sequences from the viral quasispecies dataset, with unknown fitness values, projected on this fitness landscape map. The quasispecies variants from each virus are displayed as a circle over the neuron that maps them, and the diameter of the circle symbolizes the proportion of variants identified in passage 1 (in blue), passage 11 (in green), passage 21 (yellow) and passage 31 (red). The quantification of the quasispecies variants in each neuron is summarized in Table S4 in File S1. Colour arrows joining the circles show the estimated evolutionary trajectories of the viral clones during the recovery passages.

Tha capacity of SOM networks to analyze DNA sequences not used during the training was explored by the projection of related variants into the map and the calculation of the *best matching unit* (*bmu*) for each sequence (the reference vector closest to the sequence in the map). When the SOM neurons are labeled with experimentally determined fitness, this mapping would place the sequence on a specific location of the 3D SOM fitness landscape. SOM projection of related variants was tested with members of the viral quasispecies in the V1–V2 region in *env* gene of the recovered viruses. This mapping permitted the analysis of the evolutionary recovery pathways of the different variants at the viral quasispecies level (Figure 4B).

As the quasispecies variants from the V1–V2 region had 527 nucleotides long sequences, then a new SOM network was trained and labelled with the corresponding fitness using the 527 nucleotides of the V1–V2 sequences in the 55 consensus sequences in *env* gene. The resulting fitness landscape produced by this SOM and the classification of the 55 training consensus sequences are shown in Figure 4A. Like in the phylogenetic tree and the SOM map with the complete sequences, viruses from the same lineage grouped together in the V1–V2 map, and viruses with the highest fitness (viruses H) mapped in peaks and those with low fitness in valleys (D viruses). A 0.93 Pearson correlation coeficient has been obtained by comparing the experimental fitness value of the 55 consensus sequences with the fitness value associated with the neuron that represent each sequence on the SOM map (Figure S3 in File S2). This result confirmed, again, the goodness of the graphical representation of the sequences and the experimental fitness associated to the viruses that produced the 3D SOM fitness landscape.

Next, the 911 sequences of viral quasispecies of the recovered viruses at the different passages (approximately 20 clones per virus and sample), with unknown fitness, were projected on this map. Although the map was trained with the consensus sequences, quasispecies sequences are directly related to the consensus sequences and, consequently, the projection in the map was appropriate. In fact, the quasispecies sequences mapped in the same regions than the consensus sequences (see Figures 4A and 4B). The pathways and space search of some of the variants are depicted in Figure 4B. The proportion of the different variants in each neuron of the map are summarised in Table S4 in File S1. Figure S1 in File S2 displays the Unified Distance Matrix (U-matrix) caculated for this SOM. This graph represents the Euclidean distances between the reference vectors of the SOM. In some variants, optimal evolutionary solutions are found in regions near their corresponding initial viruses, like virus *I5*, as detected in the initial landscape with the global sequences (Figure 3A). A similar behaviour was observed in virus *E1* that did not move significantly in this region along the recovery passages. In contrast, other viruses (clones *G1*, *D2* or *K1*) showed large displacements of the viral populations. U-matrix in Figure S1 in File S2 exhibited that these large displacements crossed areas of the SOM that revealed medium-high Euclidean distances. In these viruses, the generation of a large variability permitted a broader exploration of the fitness landscape to find the best evolutionary pathways. Although during the passages, some intermediate points were lost (see for example *H1.5* and *G1* at passage 11), in all cases, we noted an increase in the frequency in the population of the variants with the highest estimated fitness. These variants, in most of the viruses analysed, later became dominant (Table S4 and S5 in File S1). This can be clearly observed in virus *H* with a fitness of 0.51 in the initial passage, while in passage 11, two minor new variants were detected within a fitness peak, with an estimated fitness values of 1.57 and 2.82 (Figure 4B and Tables S4 and S5 in File S1). In posterior passages, still with a minor representation of variants from the original quasispecies, the population moved to the point with 2.82 fitness (the point with higher fitness) that later became dominant in the viral population. In summary, using SOM landscapes, we examined in detail the evolutionary processes followed by viral quasispecies along HIV-1 fitness recovery and we can analyse and approach the evolution of viral populations. Using this methodology, we were able to track the evolution of the variants of the quasispecies and to detect the fitness landscape exploration performed by each of these members of the mutant cloud.

## Discussion

Fitness landscapes provides a graphic representation which, in addition to population sizes and mutation rates, help in the understanding of evolutionary processes [9]. This report on the “*in vitro*” viral evolution in HIV-1 depicts the first fitness landscapes constructed with realistic viral data. The map was drawn with an ANN approach, in particular with a SOM algorithm, and based in the relatedness of the nucleotide sequences. This SOM map permitted the aproximation to the fitness value, based on the similarity of the sequence, of members of viral quasispecies and the visualization of the evolutionary pathways of the different HIV-1 variants during the serial passages.

The three-dimensional SOM maps are constructed first by creating a two dimensions matrix based on sequence similarity, where the experimental fitness values are included later as a third dimension. The goodness of the grouping of the sequences carried out by the SOM maps either with the complete (Figure 3A) or the consensus V1–V2 sequences (Figure 4A) is similar to the one obtained with the phylogenetic tree (Figure 2). Furthermore, minimum spannig tree analysis [29] was performed for both the complete and consensus sequences and their projection into a plane, defined by the two largest eigenvectors, illustrated the evolution of the population. These trees provided information on the quasiespecies structure as shown in Figure S4 in File S2. Clustering of viral sequences obtained by this method confirmed the grouping of sequences obtained by the SOMs (Figure 3A compared with Figure S4A in File S2 and Figure 4A with Figure S4B in File S2). In addition, the projection of the experimental fitness values as a third dimension in the SOM maps is supported by a good correlation (Figures S2 and S3). When the SOM is used to project DNA sequences related with those used in the training of the network, fitness 3D map permits an initial approach to the fitness value of a sequence, which is the fitness value associated with the neuron that identifies the sequence. Futhermore, SOM 3D maps provides important information related to the topographical characteristics of the area where sequences mapped like valleys, peaks or flat regions.

The depiction of a real fitness landscape during a fitness recovery process in HIV-1 has important advantages. First, 3D fitness landscapes allow the identification of different regions, like valleys (see *D* lineage viruses) where a few changes could lead to viral extinction (see Figure 3 and 4) or peaks (see *K* and *G* viruses). The analysis and mapping of mutations in these viruses could provide important information for genomic alterations and regions critical for viral fitness. In addition, these landscapes permitted the display during the passages of the mobilization through the landscape of viral populations and the depiction of viral evolutionary pathways. For example, clone *I* recovery trajectory showed that, although other fitness peaks are found in distant regions of the landscape, the final virus seems to return to the initial virus peak indicating that this peak could be acting like a fitness attractor (Figures 3 and 4). In viruses with low fitness before the recovery passages [4], there was a large space search and a remarkable fitness increase (viruses *G* and *D*). This search confirmed the great importance of variability generation in the exploration of the fitness landscape and in fitness increases. In contrast, other lineages displayed a more limited fitness trajectory (viruses *E*). The search in the sequence space, analyzed in computer simulations, has been classified into minor and major transitions [34]. However, in the SOM fitness maps the long or short displacements observed in the viral populations could not be directly transformed into major or minor transitions because of the non uniformity of the 2D representation as it is shown in the U-Matrix (see Figure S1 in File S2). In summary, the 3D SOM fitness landscape provided important information with the identification of abrupt zones like peaks or valleys, or the wide space search undertaken by some clones [7]. These landscapes illustrated the dynamics of the HIV-1 “*in vitro*” fitness recovery.

Once a SOM fitness landscape has been created, it could be used for the analysis of related viruses, where only nucleotide sequences are known. This relatedness means that the genetic information of the samples of study is within the boundaries of the training sequences. In general, for a more accurate analysis, the better, wider and related to the new sequences is the training set the better will be 3D SOM fitness landscape and the fitness exploration for the related sequences. The SOM maps are very useful for the visualization of the fitness landscapes, with a very short computational time, and for the identification of evolutionary viral pathways during recovery passages.

The SOM methodology can be applied for sequences from pathogenic infections by microorganisms like parasites, fungus or bacteria but especially for viruses, and, because of their enormous variation, particularly RNA viruses. SOM landscapes can be used, whenever a set of sequences is associated with a phenotypic characteristic that is sequence-dependent, for the study of the evolution of different variants. SOM maps can be employed for the analysis of complete viral genomes, individual genes or genomic fragments. SOM maps permit the fitness analysis of individual members of viral quasispecies. The use of SOM landscapes is particularly appropriate for the analysis of the enormous amount of sequences obtained from next generation sequencing (NGS) technologies. In these sequences, the experimental fitness determination of individual sequences is technically unfeasible, but it can be approximated in SOM fitness landscapes by experimentally calculating the fitness of a good representation of the different variants of the viral population.

Fitness landscapes have been used to study the evolution in theoretical works with mathemathical numerical simulation and master equations [22], also the survival of the flattest has been theoretically predicted [35]. More recently, fitness landscapes have been used to study the “*in silico*” evolution of viral quasispecies according to their mutation rate [36]. Two dimensions (2D) fitness landscapes have been represented for HIV-1 protease resistant mutant variants [20] and fitness estimated for antiviral resistant variants [20], [37]. Recently a three dimension (3D) representation of individual HIV drug resistance mutations found in field variants has been published [21], where the epistatic value of the different mutations is associated as a third dimension [21], [28], [38]. In a further study, the authors analysed the conditions for the fitness space representation [28]. In contrast, the SOM maps permit a 2D representation of viruses based on the viral sequences similarity. In this map neurons are labelled, as a third dimension, with the experimental fitness values of viruses, producing a 3D representation of a global real fitness landscape. This low-dimension representation can capture important features of the complex fitness landscapes, showing aspects related to the underlying structure of the data.

This report is the first 3D representation of an HIV-1 realistic fitness landscape using SOM. These maps allowed the understanding of the mechanisms operating during HIV-1 “in vitro” evolution, and also represented, because of the capacity of SOM to project sequences not used in the training, an innovative approach for fitness analysis of related variants. SOM fitness landscapes permitted the disclosure of viral evolutionary pathways and the inference of the potential evolution of a viral population. The capacity of the methodology, although limited to related variants, allows the characterization of individual variants with evolutionary potential within viral quasispecies that could be very helpful in *in vitro* works or *in vivo* studies. In this line, SOM fitness landscapes could have the capability for the approximation to fitness analysis and evolution.

## Supporting Information

File S1 **Tables S1–S4.**
(DOCX)

File S2 **Supporting materials and figures.** Figure S1, Unified distance Matrix for the trained SOM using viral consensus sequences in the V1–V2 region in *env* gene. Unified Distance Matrix (U-matrix) [39] is a graphical representation of the Euclidean distances between the reference vectors of the SOM. Outlined circles represent the neurons, color-scale tone inside the circle indicates the mean Euclidean distance between the reference vector of the neuron and its immediate neighbors, and the color tone of the circles without outline placed between two neighboring neurons identifies the Euclidean distance between both reference vectors. The upper left corner of the U-matrix corresponds to the upper corner of Figure 4 (the region where K15 sequence mapped), the upper right corner of the U-matrix corresponds to the right corner of Figure 4 (the area where I15 sequence is mapped). Dark blue areas represent small distances, while red areas identify the highest distances between the reference vectors of the neurons. Figure S2, Fitness correlation with the complete viral nucleotide sequences. Correlation between the fitness value predicted by the SOM (Figure 3A) and the experimental fitness value. The scatter plot shows the predicted fitness values on the y-axis and the experimental fitness values on the x-axis. Figure S3, Fitness correlation with the consensus viral nucleotide sequences. Correlation between the fitness value predicted by the SOM (Figure 4A) and the experimental fitness value. The scatter plot shows the predicted fitness values on the y-axis and the experimental fitness values on the x-axis. Figure S4, Projection of the 55 complete and consensus viral sequences using Minimum Spanning Tree (MST) analysis. The sequences have been projected onto the plane (dots) using the two eigenvectors associated with the two largest eigenvalues of the normalized covariance matrix [29]. Dots are connected by the edges obtained by calculating the minimum spanning tree, i.e., the tree which connects all the sequences with minimum total length, calculated in Hamming distance. Numbers associated with some of the MST edges represent the Hamming distance between the sequences linked by the tree branch. (A) MST obtained for the 55 complete viral nucleotide sequences. (B) MST obtained for the 55 consensus sequences in the V1–V2 region in *env* gene.
(DOCX)
