# Correction: Realistic three dimensional fitness landscapes generated by Self Organizing Maps for the analysis of experimental HIV-1 evolution

**DOI:** 10.1371/journal.pone.0098423

**Published:** 2014-05-16

**Authors:** The PLOS ONE Staff

Figures S1 – S4 were incorrectly omitted from the Supporting Information File S2. The correct File S2 can be viewed here.

## Supporting Information

File_S2. Supporting materials and figures Figure S1, Unified distance Matrix for the trained SOM using viral consensus sequences in the V1–V2 region in *env* gene. Unified Distance Matrix (U-matrix) [39] is a graphical representation of the Euclidean distances between the reference vectors of the SOM. Outlined circles represent the neurons, color-scale tone inside the circle indicates the mean Euclidean distance between the reference vector of the neuron and its immediate neighbors, and the color tone of the circles without outline placed between two neighboring neurons identifies the Euclidean distance between both reference vectors. The upper left corner of the U-matrix corresponds to the upper corner of Figure 4 (the region where K15 sequence mapped), the upper right corner of the U-matrix corresponds to the right corner of Figure 4 (the area where I15 sequence is mapped). Dark blue areas represent small distances, while red areas identify the highest distances between the reference vectors of the neurons. Figure S2, Fitness correlation with the complete viral nucleotide sequences. Correlation between the fitness value predicted by the SOM (Figure 3A) and the experimental fitness value. The scatter plot shows the predicted fitness values on the y-axis and the experimental fitness values on the x-axis. Figure S3, Fitness correlation with the consensus viral nucleotide sequences. Correlation between the fitness value predicted by the SOM (Figure 4A) and the experimental fitness value. The scatter plot shows the predicted fitness values on the y-axis and the experimental fitness values on the x-axis. Figure S4, Projection of the 55 complete and consensus viral sequences using Minimum Spanning Tree (MST) analysis. The sequences have been projected onto the plane (dots) using the two eigenvectors associated with the two largest eigenvalues of the normalized covariance matrix [29]. Dots are connected by the edges obtained by calculating the minimum spanning tree, i.e., the tree which connects all the sequences with minimum total length, calculated in Hamming distance. Numbers associated with some of the MST edges represent the Hamming distance between the sequences linked by the tree branch. (A) MST obtained for the 55 complete viral nucleotide sequences. (B) MST obtained for the 55 consensus sequences in the V1–V2 region in *env* gene.
(DOCX)

## References

[1] Lorenzo-Redondo R, Delgado S, Morán F, Lopez-Galindez C (2014) Realistic Three Dimensional Fitness Landscapes Generated by Self Organizing Maps for the Analysis of Experimental HIV-1 Evolution. PLoS ONE 9(2): e88579 doi:10.1371/journal.pone.0088579 24586344 10.1371/journal.pone.0088579 PMC3938428

