# Supplementary material for: Realistic Three Dimensional Fitness Landscapes Generated by Self Organizing Maps for the Analysis of Experimental HIV-1 Evolution
Source: PLoS One. 2014 Feb 28;9(2):e88579. doi: 10.1371/journal.pone.0088579 (PMC3938428; doi:10.1371/journal.pone.0088579)
Supplement: File S1 — Tables S1–S4. (DOCX) [file pone.0088579.s001.docx]

**Supplementary Material Tables.**

**Table S1. Viral titers, p24 production of the viruses during the recovery passages.**

|  | Passage | |  |  | |  |  | |  |  | |
| --- | --- | --- | --- | --- | --- | --- | --- | --- | --- | --- | --- |
|  | 1 | |  | 11 | |  | 21 | |  | 31 | |
| Virus | p24*^a^* | Viral Titer*^b^* |  | p24*^a^* | Viral Titer*^b^* |  | p24*^a^* | Viral Titer*^b^* |  | p24*^a^* | Viral Titer*^b^* |
| D1 | 1,3E+06^a^ | 4.2E+05^b^ |  | 2,4E+06 | 7.5E+05 |  | 2,4E+06 | 2.4E+06 |  | 1,7E+06 | 1.3E+05 |
| D1.5 | 1,0E+06 | 4.2E+05 |  | 2,5E+06 | 1.5E+05 |  | 1,8E+06 | 1.8E+06 |  | 1,6E+06 | 4.2E+04 |
| D2 | 1,1E+06 | 3.7E+05 |  | 1,1E+06 | 5.6E+05 |  | 2,1E+06 | 5.6E+05 |  | 2,0E+06 | 7.5E+04 |
| E1.5 | 1,4E+06 | 3.7E+04 |  | 1,7E+06 | 1.2E+06 |  | 2,0E+06 | 2.4E+05 |  | 1,5E+06 | 1.8E+05 |
| G1 | 1,0E+06 | 1.2E+05 |  | 1,4E+06 | 1.8E+05 |  | 2,0E+06 | 1.3E+05 |  | 7,2E+05 | 1.0E+05 |
| G1.5 | 7,0E+05 | 1.2E+05 |  | 1,8E+06 | 1.0E+05 |  | 2,1E+06 | 1.8E+06 |  | 9,7E+05 | 2.4E+04 |
| G2 | 2,7E+06 | 7.8E+04 |  | 1,9E+06 | 4.2E+05 |  | 2,6E+06 | 1.3E+05 |  | 1,1E+06 | 3.2E+04 |
| H1.5 | 2,9E+06 | 1.3E+04 |  | 1,6E+06 | 3.7E+05 |  | 2,6E+06 | 1.0E+05 |  | 9,8E+05 | 1.3E+04 |
| I1 | 1,3E+06 | 4.0E+04 |  | 3,7E+06 | 6.5E+05 |  | 2,6E+06 | 4.2E+04 |  | 2,8E+05 | 7.5E+04 |
| I5 | 1,1E+06 | 2.5E+04 |  | 1,5E+06 | 9.5E+04 |  | 2,3E+06 | 7.5E+02 |  | 6,6E+05 | 4.2E+04 |
| K1 | 2,5E+06 | 7.5E+04 |  | 2,1E+06 | 1.3E+05 |  | 2,4E+06 | 1.0E+06 |  | 8,7E+05 | 2.4E+05 |
| K2 | 1,7E+06 | 4.9E+04 |  | 2,6E+06 | 4.4E+04 |  | 1,9E+06 | 1.0E+06 |  | 9,3E+05 | 3.2E+05 |

*^a^* Viral p24 production in the supernatant of each viral culture was quantified with the Elecsys (HIV Ag, Roche) in pg per ml.

*^b^* Viral titration performed in MT-2 cells and titer expressed in TCID 50 /ml.

**Table S2. Sequence divergence between all viruses.**

Table shows all pairwise divergence values, in percentage, in the upper right part in the X axis, and per cent identity in the left Y axis, corresponding to the complete sequences of the viruses studied. Pairwise calculations between each of the sequences analysed were obtained the Megalign program (Lasergene Inc.).

**Table S3. SOM training parameters used in this work.**

Training parameters used in all the SOM networks of the study. The establishment of the parameters and the topology of a network is a heuristic problem. Based on previous experiences and related topologies, a good balance between effectiveness, resolution and computing time can be reached. In the present study, the dimension of the SOM grid has been estimated in relation to the number of cases available and selecting one map a little wider for the spread of the data.

**Table S4. Quantification of the quasispecies variants in each neuron of the SOM map during recovery passages.**

Number of the different variants in the quasispecies, arising during the recovery passages, mapping in each neuron of the 15x15 SOM map (Fig 4B). Each cell includes the name of the quasispecies variant (K1, G1, I5, D2, H1.5 and E1.5), the fitness value, and the number of quasispecies variants mapping in each neuron (passage 1 in blue, passage 11 in green, passage 21 in yellow and passage 31 in red).

**Table S5. Proportion of the different variants within quasispecies along the recovery passages.**

Presence of each quasispecies variant (in percentage) in D2, G1, H1.5, I5 and K1 clones along the recovery passages. In all lineages the fitter variant got imposed in the viral population at passage 31.
