# Supplementary material for: Correction: Realistic three dimensional fitness landscapes generated by Self Organizing Maps for the analysis of experimental HIV-1 evolution
Source: PLoS One. 2014 May 16;9(5):e98423. doi: 10.1371/journal.pone.0098423 (PMC4024037; doi:10.1371/journal.pone.0098423)
Supplement: File_S2. Supporting materials and figures — Figure S1, Unified distance Matrix for the trained SOM using viral consensus sequences in the V1–V2 region in env gene. Unified Distance Matrix (U-matrix) [39] is a graphical representation of the Euclidean distances between the reference vectors of the SOM. Outlined circles represent the neurons, color-scale tone inside the circle indicates the mean Euclidean distance between the reference vector of the neuron and its immediate neighbors, and the color tone of the circles without outline placed between two neighboring neurons identifies the Euclidean distance between both reference vectors. The upper left corner of the U-matrix corresponds to the upper corner of Figure 4 (the region where K15 sequence mapped), the upper right corner of the U-matrix corresponds to the right corner of Figure 4 (the area where I15 sequence is mapped). Dark blue areas represent small distances, while red areas identify the highest distances between the reference vectors of the neurons. Figure S2, Fitness correlation with the complete viral nucleotide sequences. Correlation between the fitness value predicted by the SOM (Figure 3A) and the experimental fitness value. The scatter plot shows the predicted fitness values on the y-axis and the experimental fitness values on the x-axis. Figure S3, Fitness correlation with the consensus viral nucleotide sequences. Correlation between the fitness value predicted by the SOM (Figure 4A) and the experimental fitness value. The scatter plot shows the predicted fitness values on the y-axis and the experimental fitness values on the x-axis. Figure S4, Projection of the 55 complete and consensus viral sequences using Minimum Spanning Tree (MST) analysis. The sequences have been projected onto the plane (dots) using the two eigenvectors associated with the two largest eigenvalues of the normalized covariance matrix [29]. Dots are connected by the edges obtained by calculating the minimum spanning tree, i.e., the tree which connects all the sequences w [file pone.0098423.s001.docx]

**Supplementary Material:**

**Nucleotide codification method**

For the SOM algorithm, two issues are critical: the calculation of distances between the sample and the reference vectors of the map, and the stepwise approximation of the reference to the sample vectors. In the determination of the network reference-vector closest to an input vector, SOM utilizes Euclidean distances. This need represents a problem with non-numerical data vectors like nucleotide sequences (DNA or RNA) (S1-S4). This problem can be overcome by the transformation of the sequences into numerical vectors. The codification method that we propose is a necessary and simple strategy to convert a nucleotide sequence into a numerical vector. With this numerical vector we can apply the SOM training algorithm. Basically, the method permits the adaptation of the synaptic vectors that have to be carried out by small approaching steps or by small modifications of components of the synaptic vectors. This process cannot be performed directly with nucleotide sequences represented by letters.

The nucleotide sequence codification method transforms Adenine, Guanine, Cytosine and Thymine into the 3-D coordinates of one of the four vertices of a 3-dimensional irregular tetrahedron, with distance 1 between A-G and C-T vertices, and distance 2 for all other combinations. Here we consider more probable AG and CT transitions than AC and GT transversions. As a requirement of SOM, sequences must be of identical length, and insertions and deletions are counted and encoded in the centre of the tetrahedron in the 3D-coordinates. Once DNA sequences are transformed into the corresponding coordinates-vector, the distances between sequences are calculated in a 3*N dimensional real space, where N is the length of the sequences, in terms of Euclidean distances. In this way, the topologic similarity is defined as “Euclidean similarity” between sequences.


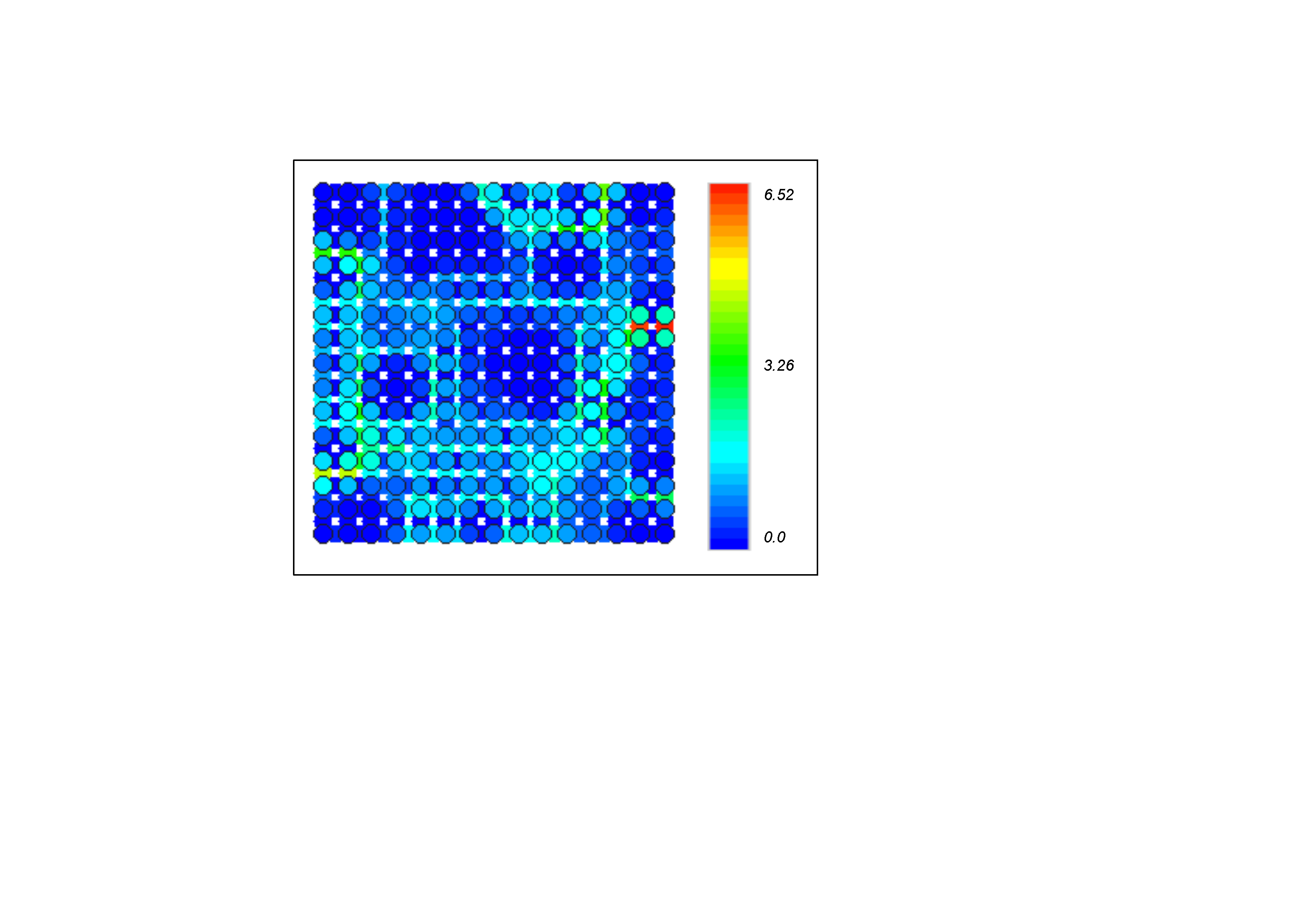


**Figure S1. Unified distance Matrix for the trained SOM using viral consensus sequences in the V1‐V2 region in *env* gene.**

Unified Distance Matrix (U-matrix) [[39](#_ENREF_39)] is a graphical representation of the Euclidean distances between the reference vectors of the SOM. Outlined circles represent the neurons, color-scale tone inside the circle indicates the mean Euclidean distance between the reference vector of the neuron and its immediate neighbors, and the color tone of the circles without outline placed between two neighboring neurons identifies the Euclidean distance between both reference vectors. The upper left corner of the U-matrix corresponds to the upper corner of Figure 4 (the region where K15 sequence mapped), the upper right corner of the U-matrix corresponds to the right corner of Figure 4 (the area where I15 sequence is mapped). Dark blue areas represent small distances, while red areas identify the highest distances between the reference vectors of the neurons.


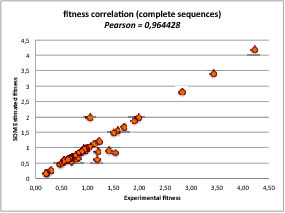


**Figure S2. Fitness correlation with the complete viral nucleotide sequences.** Correlation between the fitness value predicted by the SOM (Figure 3A) and the experimental fitness value. The scatter plot shows the predicted fitness values on the y-axis and the experimental fitness values on the x-axis.

**
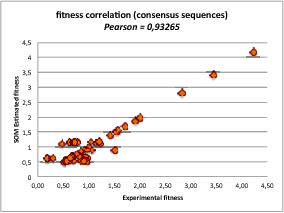
**

**Figure S3. Fitness correlation with the consensus viral nucleotide sequences.** Correlation between the fitness value predicted by the SOM (Figure 4A) and the experimental fitness value. The scatter plot shows the predicted fitness values on the y-axis and the experimental fitness values on the x-axis.

**
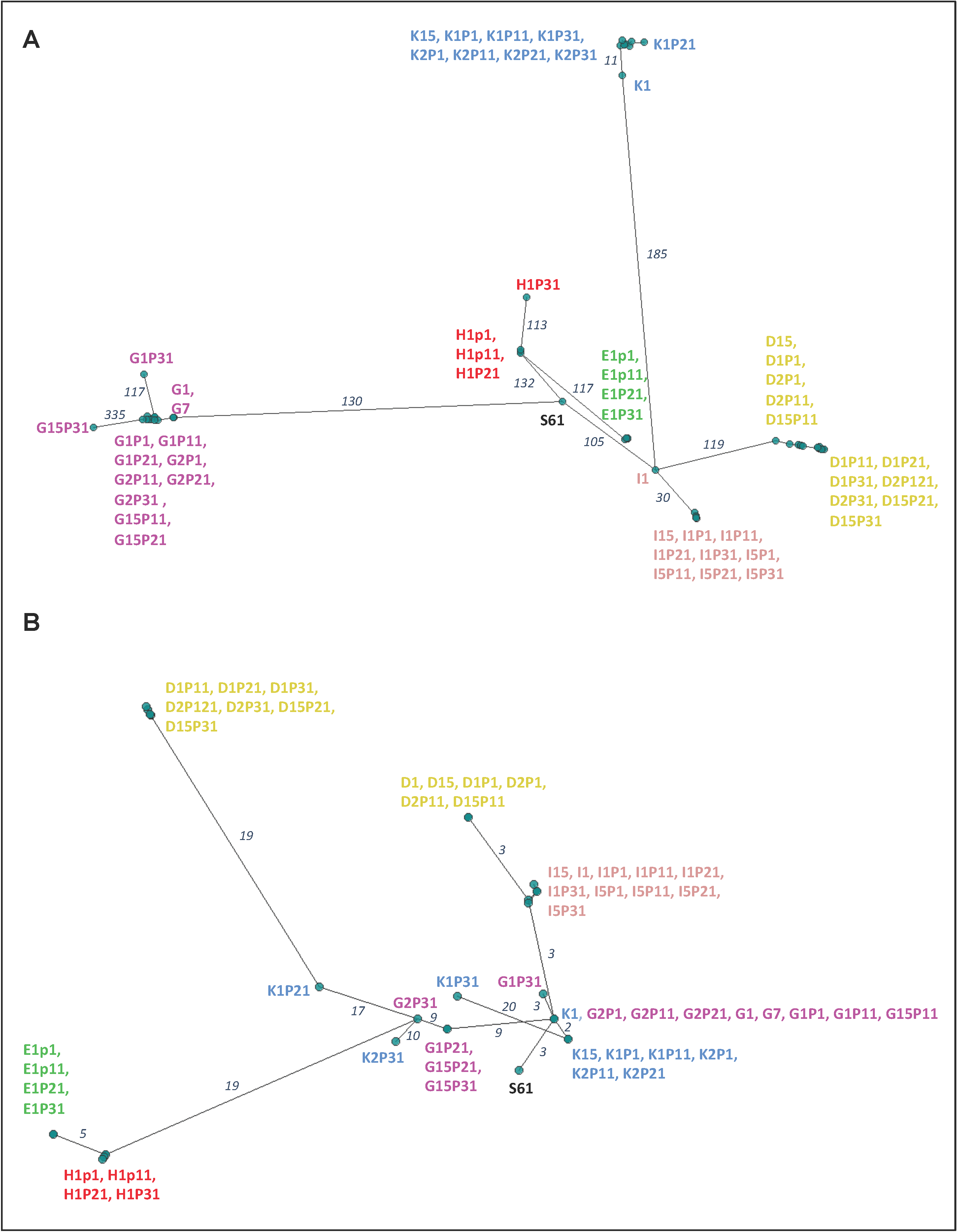
**

**Figure S4. Projection of the 55 complete and consensus viral sequences using Minimum Spanning Tree (MST) analysis.**

The sequences have been projected onto the plane (dots) using the two eigenvectors associated with the two largest eigenvalues of the normalized covariance matrix [[29](#_ENREF_29)]. Dots are connected by the edges obtained by calculating the minimum spanning tree, i.e., the tree which connects all the sequences with minimum total length, calculated in Hamming distance. Numbers associated with some of the MST edges represent the Hamming distance between the sequences linked by the tree branch. (A) MST obtained for the 55 complete viral nucleotide sequences. (B) MST obtained for the 55 consensus sequences in the V1-V2 region in *env* gene.

**SUPPLEMENTARY REFERENCES.**

S1. **Kohonen, T., and P. Somervuo**. 2002. How to make large self-organizing maps for nonvectorial data. Neural Netw **15**:945-952.

S2. **Andrade, M. A., G. Casari, C. Sander, and A. Valencia**. 1997. Classification of protein families and detection of the determinant residues with an improved self-organizing map. Biol Cybern **76**:441-450.

S3. **Ferran, E. A., and P. Ferrara**. 1991. Topological maps of protein sequences. Biol Cybern **65**:451-458.

S4. **Gatherer, D**. 2007. Genome signatures, self-organizing maps and higher order phylogenies: a parametric analysis. Evolutionary bioinformatics online **3**:211-236.
